# Acute respiratory distress syndrome as the initial symptom of hepatic angiosarcoma with Kasabach–Merritt syndrome: A case report

**DOI:** 10.1097/MD.0000000000039800

**Published:** 2024-09-27

**Authors:** Xiaoqian Cui, Min Zhang, Debiao Song, Jiakun Tian

**Affiliations:** a Department of Critical Care Medicine, The Second Hospital of Jilin University, Changchun, Jilin, China.

**Keywords:** acute respiratory distress syndrome (ARDS), case report, hepatic angiosarcoma (HSA), Kasabach–Merritt syndrome (KMS), oncology

## Abstract

**Rationale:**

Hepatic angiosarcoma (HSA) has a poor prognosis. Our understanding of its clinical features, diagnosis, treatment, and prognosis remains limited. In certain cases, vascular tumors such as HSA can induce bleeding complications due to thrombocytopenia, known as Kasabach–Merritt syndrome (KMS). When KMS symptoms occur in the lungs, its clinical manifestations closely resemble those of ARDS, leading to misdiagnosis and poor outcomes. Unfortunately, this condition is extremely rare and there is a lack of relevant case reports, which further adds to the difficulty of its diagnosis and treatment.

**Patient concerns:**

This case report describes a patient who initially presented with symptoms of ARDS. Due to the unique nature of these symptoms, the patient underwent a complex diagnostic and treatment process before finally being diagnosed with HSA complicated by KMS through pathological examination.

**Diagnoses:**

The patient was eventually diagnosed with HSA by pathology and KMS with multiorgan hemorrhage.

**Interventions:**

Highly misleading clinical manifestations were recorded during the diagnosis and treatment, which, to our knowledge, have not been previously reported.

**Outcomes:**

The patient died from a massive pulmonary hemorrhage.

**Lessons:**

Dysfunction of a single organ or system may be the external manifestation of a multi-system clinical disease. Therefore, in the clinical diagnosis and treatment process, especially during early diagnosis, while it is important to focus on the primary or typical clinical symptoms, it is equally crucial not to underestimate or ignore accompanying symptoms that lack specificity. When diagnosis and treatment reach an impasse, these “atypical” symptoms often prove to be key in solving the puzzle.

## 1. Introduction

Hepatic angiosarcoma (HSA) is a sporadic tumor originating from endothelial cells in the liver. It represents approximately 5% of all angiosarcomas and accounts for 0.1% to 2% of liver malignancies. It is characterized by highly aggressive behaviors and rapid progression.^[1,2]^ Reports indicate that among the postoperative pathological results of 1500 cases of surgical resection for adults’ primary hepatic tumors, only 2 cases were confirmed to be HAS.^[3]^ Unfortunately, HSA is often misdiagnosed or overlooked due to the absence of typical clinical symptoms or specific markers. Most patients lack typical symptoms. According to reports, when the tumor exceeds 5 cm in size, patients may experience abdominal discomfort or pain. However, even at this stage, HSA still does not present significant abnormalities in laboratory indicators.^[4]^ A definitive diagnosis requires a pathological examination of the biopsy tissue.^[5]^ The main treatment methods include surgery, adjuvant chemotherapy, transcatheter embolization (TAE), and transarterial chemoembolization. In some reports, teams have attempted to treat HSA using partial hepatectomy or liver transplantation. However, the postoperative survival period of patients often does not exceed 1 year.^[3,6]^ HSA has an overall poor outcome. Despite existing studies and case reports,^[7,8]^ the understanding of the disease’s clinical characteristics, diagnostic criteria, treatment strategies, and prognosis remains limited.

Kasabach–Merritt syndrome (KMS) was first described in a case of severe thrombocytopenia in an infant with giant hemangioma.^[9]^ KMS can be divided into 2 stages based on the progression: the coagulopathy stage and the residual lesion stage. The main manifestations of the coagulopathy stage include anemia, thrombocytopenia, elevated D-dimer level, decreased fibrinogen level, and increased number of fibrin degradation products.^[10]^ In the few reported cases of KMS in children, when KMS symptoms occur in the lungs, the clinical manifestation include chest tightness, shortness of breath, bloody sputum, and even dyspnea, which are closely resemble ARDS.^[11,12]^ KMS in adults is even rarer, often caused by metastatic vascular malignancies.^[13]^ In adult patients, KMS caused by PHA is even rarer, with only case reports available,^[14]^ making it impossible to calculate its specific incidence rate. In a report including 5 cases of HSA-induced KMS, patients experienced improvement in coagulation function after tumor resection surgery. However, these patients did not present with significant pulmonary symptoms, only exhibiting coagulation abnormalities.^[4]^ However, to date, no adult KMS cases with initial pulmonary symptoms have been reported.

Acute respiratory distress syndrome (ARDS) is an acute respiratory failure caused by multiple pulmonary diseases (pulmonary infection and inhalation disease) or nonpulmonary diseases (sepsis, pancreatitis, and trauma). It is characterized by acute inflammatory lung injury, resulting in increased pulmonary capillary permeability. The clinical manifestation is hypoxemia.^[15]^ The diagnosis of ARDS is based on the Berlin definition criteria for the timing of the syndrome’s onset, origin of edema, chest radiograph findings, and hypoxemia.^[16]^ In most cases, infection-related ARDS can be differentiated from KMS by investigating the present history and radiographic results. However, in rare cases, these 2 diseases can exhibit highly similar performance.

In the present case, the patient presented with a combination of ARDS and KMS. The diagnosis was misleading and problematic due to the rare initial symptoms and presentations of this case. The patient was eventually diagnosed with HSA by pathology and died of unmanageable massive hemoptysis. Highly misleading clinical manifestations were recorded during the diagnosis and treatment, which, to our knowledge, have not been previously reported. The case aimed to improve the understanding of this disease, identify atypical clinical features and warn clinicians about potential misleading factors.

## 2. Case presentation

### 2.1. Initial presentation

On October 25, 2022, a male patient in his early 70s was admitted with intermittent left shoulder and presternal pain for 3 days, worsening with intermittent syncope in the last 1 day. The patient had a history of coronary heart disease and underwent coronary stent implantation 8 years ago. Also, the patient had a 30-year history of chronic hepatitis B virus infection, treated with long-term entecavir antiviral therapy, and a 5-year history of chronic hepatitis C virus infection, for which he had received a 12-week course of sofosbuvir/velpatasvir direct-acting antiviral therapy. Unfortunately, the patient did not undergo a liver CT examination at that time. In the regularly conducted liver function monitoring, the patient’s liver function (transaminases, bilirubin, etc.) did not show significant abnormalities. The most recent liver function test was performed 6 months prior to this admission. As the coagulation tests on admission revealed significantly elevated levels of D-dimer and fibrin degradation products, the emergency physician suspected pulmonary embolism, myocardial infarction, or transient ischemic attack based on the symptoms. To clarify the cause, the patient underwent head magnetic resonance imaging (MRI) and echocardiography on the day of admission. Head MRI did not reveal any definitive positive findings, whereas echocardiography showed left ventricular diastolic dysfunction and hypokinesia. The patient underwent CT pulmonary angiography (CTPA) and CT coronary angiography on hospital day 2 to further determine the etiology. CT coronary angiography demonstrated mild-to-moderate stenosis (10–30%) of the left anterior descending, left circumflex, and right coronary arteries. The coronary stents were unblocked. CTPA did not reveal pulmonary artery stenosis. Bilateral multifocal ground-glass opacities and bilateral subpleural reticulations were observed on the chest CT. However, the CT findings were interpreted as mild pulmonary inflammation without targeted treatment or further workup. The patient received supplemental oxygen, monitoring, and supportive therapy after admission. His symptoms improved significantly with rest and oxygen therapy. Subsequently, he was discharged on October 26 and advised to follow up with his physician. The working diagnoses at discharge were pneumonia and transient ischemic attack.

### 2.2. Disease progression

On November 14, 2022, this patient was again brought to the emergency department by his family, now with chest tightness, dyspnea, and abdominal discomfort for 8 days. Due to significant dyspnea, the patient underwent chest high-resolution computed tomography and a second CTPA in the emergency department. The results showed new multiple pulmonary nodules, and the ground-glass opacities significantly increased compared with those on October 26. Pulmonary embolism was again excluded. The patient was transferred to the intensive care unit (ICU) 4 hours after presentation to the emergency department due to progressive respiratory distress. The working diagnosis at this time was severe pneumonia.

**Figure 1.**
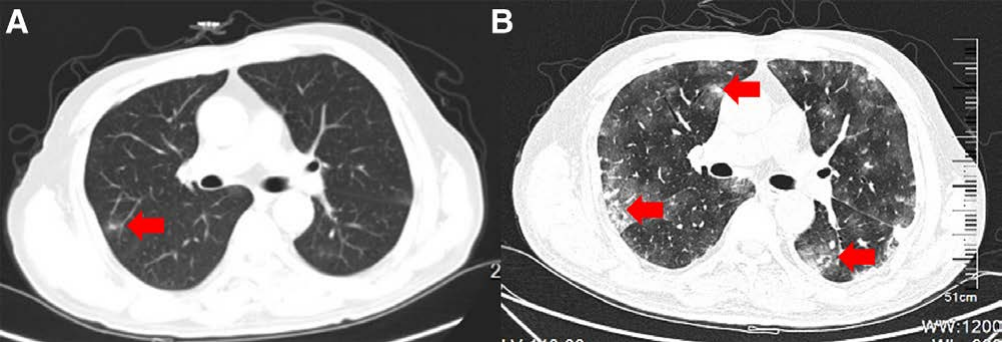
On October 26, a chest CT showed newly formed ground-glass opacities (A). On November 14, multifocal nodular high-density shadows and patchy ground-glass opacities were observed in both lungs (B). As indicated by the red marks, at the same level, both the extent of the lesion and the severity of pulmonary edema have significantly worsened between the 2 CT examinations. CT = computerized tomography.

The arterial blood gas analysis at the ICU bedside showed type I respiratory failure. The arterial partial pressure of oxygen (PaO_2_) decreased to 58 mm Hg, the arterial partial pressure of carbon dioxide (PaCO_2_) to 34 mm Hg, and the oxygenation index to 128 mm Hg. The results of other laboratory tests performed upon ICU admission are presented in Table 1. The patient immediately received high-flow oxygen therapy, intensive monitoring, and supportive care due to significantly elevated infection markers. The sputum samples were collected for culture and next-generation sequencing (NGS). Given the evidence of pulmonary edema on CT (Fig. 1) and progressive dyspnea, the team’s working diagnosis was ARDS caused by pulmonary infection. Based on this, empiric antibiotic therapy (piperacillin–tazobactam and voriconazole) and high-flow oxygen therapy (FiO_2_ 60%) were initiated. As the patient’s symptoms were primarily centered around the respiratory system, the ICU treatment team believed that the diagnosis of ARDS caused by pulmonary infection was essentially established. They considered that what needed to be further clarified was the type of pathogen and the strategy for anti-infective treatment. However, looking back at the outcome, an important symptom of the patient at this time - abdominal discomfort - was overlooked. At that time, the treatment team attributed this symptom to poor eating and stress response, and simply provided symptomatic treatment with antacid medications.

**Table 1:** Laboratory tests on ICU admission.

Hematology	
Hemoglobin	86 g/L (130–175 g/L)
Platelet	95 × 109/L (125–350 × 109/L)
Fibrinogen	1.54 g/dL (2.0–4.0 mg/dL)
Fibrinogen degradation product	127.6 μg/mL (0–5 μg/mL)
D-dimer	46.52 (0–1 μg/mL)
Tumor markers	
Carcinoembryonic antigen	0.92 ng/mL (0–5.0 ng/mL)
α-fetoprotein	4.12 ng/mL (0–8.78 ng/mL)
Indicator of infection	
Antibodies to hepatitis B and C viruses	Positive
Procalcitonin	0.09 ng/mL (0–5)
NGS (bronchoalveolar lavage fluid)	Acinetobacter baumannii infection

ICU = intensive care unit, NGS = next-generation sequencing.

**Figure 2.**
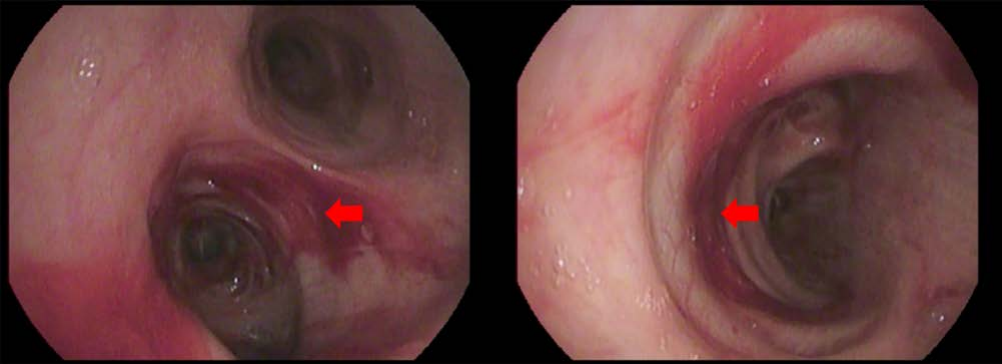
Wide-ranged bleeding in air passage was found during the bronchoscopic examination.

On November 15, the patient underwent bronchoscopy to further clarify the diagnosis and identify pathogens (Fig. 2), revealing diffuse blood-tinged exudates and scattered bleeding spots on the bronchial mucosa (possibly related to thrombocytopenia and coagulopathy). However, the cytology smears and pathology from bronchial samples were unrevealing, only excluding malignancy, tuberculosis, and fungal infection in the bronchial cavity. By now, the patient had received 2 days of antibiotic therapy, yet the infection markers continued to rise. Dyspnea seemed to alleviate, but chest tightness and abdominal pain persisted. Serial arterial blood gas monitoring showed some improvement in respiratory failure, which was likely related to high-flow oxygen. However, the patient developed gum bleeding after brushing their teeth, along with scattered petechiae on the trunk, axillae, groin, and lower extremities. On November 16, NGS reported *Acinetobacter baumannii* infection, which was susceptible to the current antibiotics. Therefore, the anti-infective regimen remained unchanged, whereas antifungal therapy was continued to prevent possible fungal co-infection. The working diagnoses at this time were severe pneumonia, ARDS, and coagulopathy. At this stage, the patient’s abdominal discomfort persisted without significant worsening, thus still failing to draw the attention of the treatment team. The newly emerged bleeding symptoms were not associated with the abdominal discomfort either, but were instead treated as a new manifestation induced by infection. This led the diagnostic and treatment process into a stalemate.

### 2.3. Establishment of HSA diagnosis

**Figure 3.**
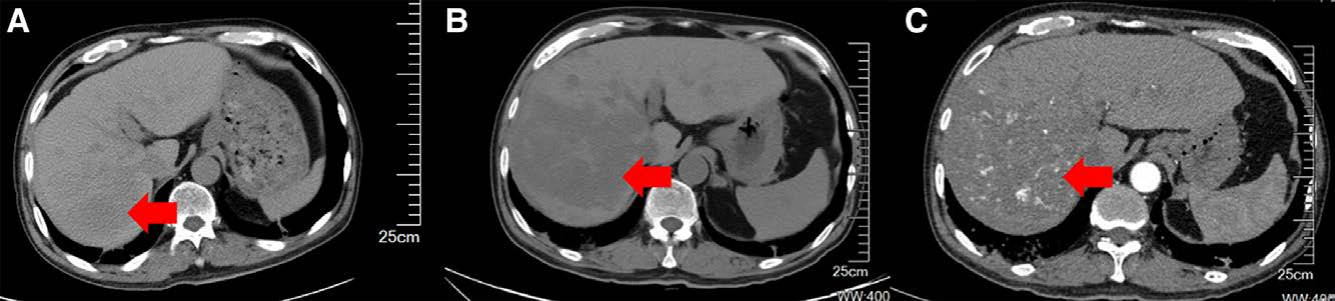
Low-density lesions were identified in the liver on October 26 (A), and these lesions showed a significant enlargement on November 14 (B and C).

**Figure 4.**
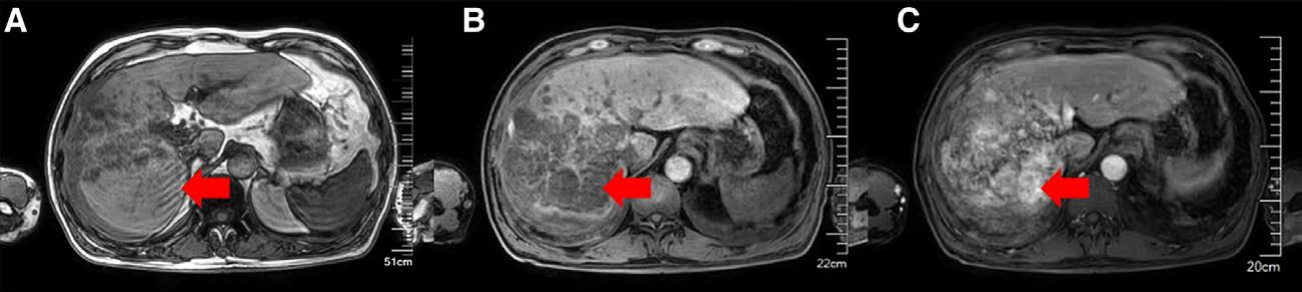
MRI examination on November 17 showed that the surface of the liver was rough with multifocal masses of T1 and T2 signal intensities. The right lobe of the liver exhibited enhancement (A), while the venous phase showed uneven enhancement, which was higher than that in the liver parenchyma (B). The arterial phase exhibited a tubular enhancement shadow proximal to the artery (C). MRI = magnetic resonance imaging.

**Figure 5.**
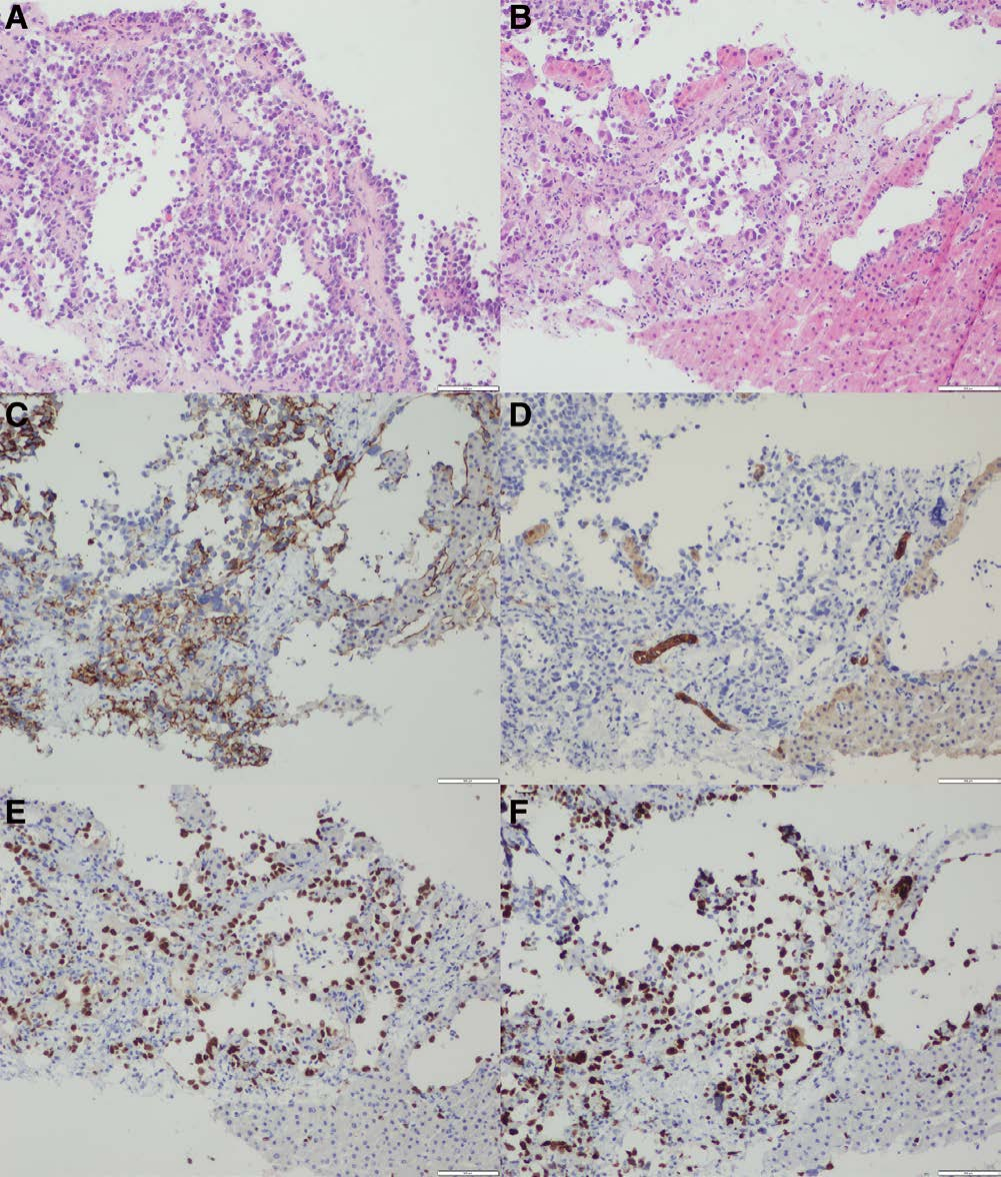
H&E and immunohistology results of the biopsy specimen: HE (A and B), CD31 (C), CK (D), ERG (E), and Ki61 (F).

On November 17, the patient’s condition still did not show fundamental improvement The abdominal pain was unrelieved, and abdominal distension and poor appetite developed. The hemoglobin level and platelet count continued to decrease, D-dimer levels increased, the number of fibrin degradation products increased, and the fibrinogen level decreased (Table 2). A multidisciplinary consultation was organized with pulmonologists and cardiologists to review the patient’s information since October 25. During the discussion, 1 member noticed the gradually worsening abdominal symptoms and reviewed the patient’s CT scans taken after admission. They discovered low-density occupying lesions in the right hepatic lobe on chest CT images taken on October 26. As the focus then was on the lungs, the radiologist interpreted these as benign hemangiomas. However, the right hepatic lesions were significantly enlarged on the chest CT taken on November 14 (Fig. 3). Considering the patient’s history of hepatitis B and cirrhosis, the hepatic surgeons and oncologists joined the team. The patient underwent a contrast-enhanced MRI of the liver and an ultrasound-guided liver biopsy. MRI showed an irregular liver surface, with multifocal masses of heterogeneous T1 and T2 signal intensities. The arterial phase displayed tubular enhancement near the hepatic artery in the right lobe. The venous phase revealed uneven enhancement, which was higher than that of the liver parenchyma. The lesion enhancement decreased slightly in the equilibrium and delayed phases, with tubular unenhanced areas showing specific hepatobiliary hypointensity (Fig. 4). The biopsy results were positive for endothelial cell markers, including CD31, ERG, and INI-1, and negative for CK, CK8/18, S-100, and TTF-1. The Ki-67 proliferative index was 60% (Fig. 5). Based on these results, the team finally established a diagnosis of HSA. At this point, the treatment team finally realized that the previously overlooked abdominal symptoms were likely the main reason for the patient’s rapid and uncontrollable progression of ARDS. Additionally, this could also be the cause of the patient’s coagulation dysfunction symptoms.

**Table 2:** Laboratory tests during treatment.

Date	Nov. 15	Nov. 16	Nov. 17	Nov. 18	Nov. 19
Leukocyte (10^9^ g/L)	13.1	13.7	12.4	13.6	20.3
Hemoglobin (g/L)	86	80	67	97	86
Platelet (/L)	95	87	70	71	64.7
D-dimer (μg/mL)	46.52	47.44	40.17	44.43	53.36
Fibrinogen (mg/dL)	1.54	1.48	1.92	1.30	0.87
Fibrinogen degradation product (μg/mL)	127.6	144.9	137	149.4	167.2

### 2.4. Rapid deterioration

**Figure 6.**
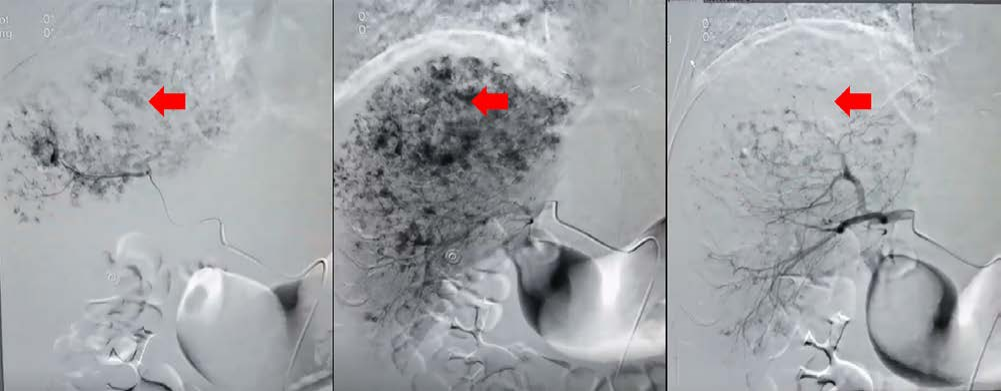
A hemorrhagic spot was identified and embolized during the endovascular intervention.

However, after the liver biopsy, the patient developed progressive pallor, restlessness, diaphoresis, and other signs of acute shock. Emergency bedside abdominal ultrasound revealed anechoic fluid in the peritoneal cavity. Diagnostic paracentesis under ultrasound guidance yielded bloody peritoneal fluid. The coagulation results also showed progressive consumption of coagulation factors. Based on these findings, the multidisciplinary team considered that the patient had a ruptured hemorrhage from the malignant neoplasm. While receiving supportive care with blood transfusion, coagulation factor supplement, and hemostatic medications, the patient underwent emergency interventional radiology embolization of the hepatic bleeding sites (Fig. 6). However, he remained hemodynamically unstable, requiring vasopressor support to maintain blood pressure despite massive transfusion.

On November 18, the patient developed a worsening cough with hemoptysis. Abdominal pain and distension persisted, with large amounts of bloody fluid draining from the peritoneal catheter. Vasopressor support with norepinephrine was still required to maintain blood pressure. The arterial blood gas analysis showed progressive respiratory failure, with the oxygenation index decreasing to 60 mm Hg. The hemoglobin level and platelet count increased slightly, which was due to transfused packed red blood cells, plasma, and platelets. The number of fibrin degradation products remained elevated, whereas the fibrinogen level continued to decrease (Table 2). The patient was now on 100% FiO_2_ with mechanical ventilation. At this point, the patient had a pulmonary hemorrhage, abdominal hemorrhage, and shock.

### 2.5. Establishment of KMS diagnosis and the death of the patient

On November 19, these problems continued to worsen. The lab tests indicated further consumption of hemoglobin, platelets, and fibrinogen despite massive transfusion (Table 2). A multidisciplinary team, including hepatic surgeons, hematologists, pulmonologists, and ICU physicians, convened again, concluding that the patient now had Kasabach–Merritt syndrome (KMS) with multiorgan hemorrhage. In light of the current situation, only supportive care to stabilize vital signs and component transfusion could be offered. On November 21, the patient died from a massive pulmonary hemorrhage.

## 3. Discussion

ARDS is a clinical syndrome characterized by refractory hypoxemia and caused by intrapulmonary and/or extrapulmonary factors. The main clinical manifestations include acute onset, respiratory distress, and hypoxemia, which is refractory to conventional oxygen measures.^[17]^ The common symptoms include shortness of breath, respiratory distress, chest tightness, cough, bloody sputum, and other symptoms. The pathogenesis of ARDS is multifactorial. The most common trigger is pulmonary inflammation. Common etiologies include bacteria, viruses, fungi, atypical pathogens, and malignant tumors.^[15]^ It is worth noting that disseminated intravascular coagulopathy (DIC) or coagulation disorders caused by other factors have also been proven to induce ARDS by promoting the formation of widespread microthrombosis in pulmonary capillaries.^[18,19]^ Lung CT may show ground-glass opacities in the early stage of ARDS and unevenly distributed lesions from the anterior to posterior borders of the lungs in the terminal stage.^[20]^ Taken together, ARDS has diverse pathogenesis and varied radiological and pathological manifestations requiring comprehensive clinical and diagnostic evaluations and management.

HSA is an aggressive and rare tumor accounting for only 2% of all primary hepatic malignancies.^[2]^ HSA occurs in association with known chemical carcinogens, but the etiology in 75% of the patients remains unknown. Patients with hepatitis B are prone to develop cirrhosis and liver cancer subsequently.^[21,22]^ A more common and often overlooked link is between liver fibrosis and cirrhosis. Cirrhosis has been reported in 42.3% of HSA biopsy specimens.^[23]^ The clinical symptoms of HSA are not typical. Patients with HSA commonly present with nonspecific symptoms such as abdominal pain, weight loss, fatigue, or an abdominal mass. Some patients may also experience symptoms of liver disease, such as nausea, vomiting, malaise, weakness, anorexia, and fever. In summary, HSA lacks characteristic clinical manifestations, and patients often present with nonspecific symptoms leading to delayed diagnosis or misdiagnosis.

Achieving accurate diagnosis is challenging due to the lack of specific clinical manifestations in patients with HSA. Some clinicians misdiagnosed HSA as hepatic cysts, hepatic abscesses, hepatic adenoma, cholangiocarcinoma, gallstones, and spindle cell tumors.^[2]^ Serial radiological imaging may demonstrate the rapid growth of the tumor, and MRI features can be more informative than CT findings. The value of PET imaging can also be illuminating.^[24]^ The liver biopsy is the gold standard for establishing the diagnosis of HSA. Considering the high bleeding tendency of HSA, some reports suggest that fine-needle aspiration cytology may be a relatively safe choice.^[21]^ Generally, the immunohistochemical staining for HSA is positive for vascular endothelial cell markers, including CD34, CD31, and factor VIII–related antigens. Other positive markers are vimentin, ERG, GPC-3, desmin, and so on. Among these, CD31 and ERG are considered sensitive endothelial marker, but they are not specific.^[25–27]^

HSA has a dismal prognosis. The median overall survival is only 5 months.^[28]^ The factors portending a poor prognosis are older age, large tumor size, and a high Ki-67 proliferation rate.^[29]^ However, no established guidelines or standard treatment consensus exists for the optimal management of HSA. The main treatment methods include surgery, adjuvant chemotherapy, TAE, and transarterial chemoembolization.^[30]^ In addition, liver transplantation is precluded by a high recurrence risk. Complete surgical resection is the only definitive treatment that can improve survival. However, certain patients are unresectable after tumor evaluation once they are found.

KMS was first reported in 1940 as thrombocytopenia associated with giant hemangioma in pediatric patients. It is a rare complication of vascular tumors, which is usually found in children with kaposiform haemangioendothelioma or tufted angioma. However, it may also occur in adults with other vascular neoplasms such as HAS in the present case.^[31]^ This syndrome is now used to describe thrombocytopenia and consumptive coagulopathy associated with vascular tumors. Previous studies also indicated that the age of presentation, morphology, and tumor size were independent risk factors for KMS.^[32]^ Existing theories suggest platelet aggregation in hemangioma in which malignant endothelial cells further promote platelet activation and adhesion to trigger KMS development.^[13]^ The abnormal proliferation of vascular endothelial cells in the lesion can capture platelets; promote platelet adhesion, aggregation, and activation leading to activation of the coagulation cascade. The fibrinolytic system is hyperactive, resulting in intratumoral hemorrhage, rapid tumor enlargement, and a new round of the consumption of coagulation substances. In severe cases, it can potentially lead to disseminated intravascular coagulation. The progressive worsening of thrombocytopenia, together with disseminated intravascular coagulation and hypofibrinogenemia, eventually results in intralesional bleeding.^[32]^ Recent studies suggest that KMS is essentially a manifestation of DIC. Through the aforementioned mechanism, coagulation factors are extensively consumed within the tumor, subsequently affecting the entire body and leading to symptoms, ultimately resulting in the occurrence of KMS.^[33,34]^ The presenting symptoms in the few reported cases include abdominal pain, increased mass size, disseminated intravascular coagulopathy, bleeding, fractures, and high-output cardiac failure. The laboratory findings of consumptive coagulopathy can also be referenced.^[35,36]^ No clear guidelines for diagnosis and management are available owing to the rarity of this syndrome. Known treatment methods include tumor resection, pre-operative and post-operative adjuvant chemotherapy, glucocorticoid therapy as well as infusion of coagulation factors and plasma to address coagulation disorders.^[4,12,37–39]^

Reports of KMS in adults are extremely rare, and in most reported cases, patients did not have the opportunity to receive effective treatment. Similar to pediatric KMS, adult KMS is also often secondary to vascular neoplasms. In a case report from 2016,^[13]^ a patient with a history of malignant tumor developed KMS after metastasis occurred. In this case, the symptoms of KMS developed gradually. Additionally, due to the patient’s history of malignant tumor, changes in coagulation function were noticed early in the onset of the disease. Eventually, this patient’s KMS symptoms significantly improved after receiving chemotherapy with gemcitabine and vinorelbine, allowing for continued routine chemotherapy. This is one of the few reports we found of adult KMS receiving subsequent treatment. Compared to the aforementioned case, our reported case has several unique features: Firstly, the medical history was unclear. The patient was hospitalized for infectious ARDS, and no history of malignant tumors was discovered before admission. This initially led the medical team in the wrong direction. Secondly, the patient’s coagulation function abnormalities appeared gradually and progressively worsened during treatment. Due to the initial misdiagnosis, this presentation was considered a sign of worsening infection. In the later stages of diagnosis and treatment, the coagulation dysfunction caused by KMS showed rapid progression, ultimately leading to the patient’s death. Lastly, among the many symptoms, the only 1 that might have pointed towards the correct diagnosis - abdominal discomfort - was only noticed late in the diagnostic process due to its lack of specificity.

**Figure 7.**
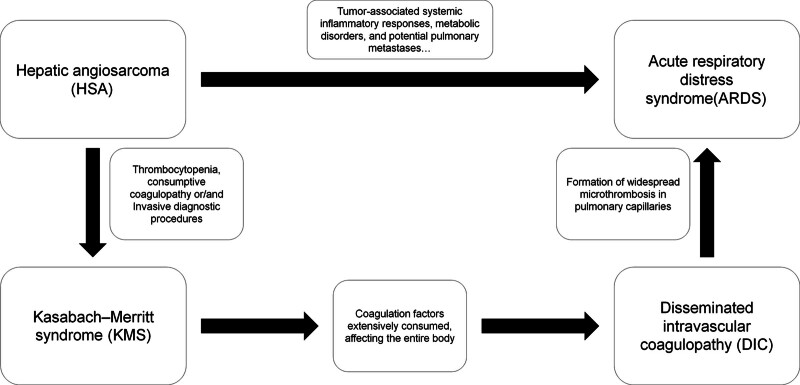
Presumed possible link between HAS, KMS and ARDS. ARDS = acute respiratory distress syndrome, DIC = disseminated intravascular coagulopathy, HSA = hepatic angiosarcoma, KMS = Kasabach–Merritt syndrome.

Currently, there is no research that clearly elucidates the relationship among HSA, KMS, and ARDS. However, based on case reports with relevant symptoms that we have retrieved, we can speculate on the mechanism of occurrence. The first pathway is relatively more direct, HSA, as a rapidly progressing and highly invasive malignant tumor, inherently affects multiple organs throughout the body. Examples include tumor-associated systemic inflammatory responses, metabolic disorders, and potential pulmonary metastases. These conditions can trigger respiratory distress or even ARDS in patients before they manifest KMS-related symptoms.^[38]^ Valenzuela et al reported such a case in 2009,^[40]^ where a 65-year-old male patient was admitted due to abdominal tension, jaundice, and fatigue. His condition rapidly deteriorated, presenting with ARDS, and he ultimately died 36 hours after admission. The patient was eventually diagnosed with HSA through autopsy, without exhibiting KMS symptoms during treatment. Another pathway is more complex. KMS may further trigger DIC following HSA, inducing ARDS through the mechanism of widespread pulmonary capillary microthrombosis mentioned before. The biopsy we performed to further clarify the diagnosis may have accelerated this process, leading to the rapid deterioration of the patient’s condition. Karaböcüoğlu et al^[37]^ reported a similar situation where a needle aspiration of the local lesion accelerated the progression of KMS, resulting in systemic multi-organ symptoms, including respiratory distress, within a short period. In this case, the patient was immediately transferred to the ICU after the onset of symptoms. Based on symptomatic supportive treatment, the patient received continuous infusion of coagulation factors, plasma, and glucocorticoid therapy. The patient’s condition improved after 1 week. This is also the only case we have found to date where a patient’s symptoms improved after treatment following KMS induced by needle biopsy. Figure 7 shows the presumed possible mechanism.

The initial symptoms experienced by this patient, including chest tightness, shortness of breath, and progressive difficulty breathing, were consistent with the features of ARDS. Combined with the findings of CT examination and bronchoscopy, we believe that this is a typical case of infection-induced ARDS. This conclusion greatly misleads our medical thinking. After targeted anti-infection, oxygen therapy and symptomatic treatment, the patient’s symptoms did not get fundamental improvement, but showed a trend of further aggravation, and began to appear abnormal coagulation function. This forced us to review the patient’s examination results and found evidence of liver tumor on the chest CT. It is now presumed that at this point the patient has developed KMS symptoms caused by HSA. However, due to the lack of typical clinical manifestations of KMS, we are still unable to make a definitive diagnosis at this time. Finally, in the case of progressive aggravation of the patient’s condition, in order to obtain a definitive diagnosis, we took the risk to perform a fine needle puncture pathology examination and obtained a pathological diagnosis of HSA. Considering the high bleeding tendency of HSA, we actively corrected the patient’s coagulation dysfunction through transfusions of platelets, clotting factors, and plasma during treatment. The patient’s coagulation function, hemoglobin level, and ascites post-biopsy were closely monitored. At the same time, an interventional team was on standby to perform emergency embolization if bleeding occurred. We hoped that these measures might help us minimize the risks of biopsy and start further treatments, including chemotherapy, as soon as possible after a definitive diagnosis. Unfortunately, hemorrhage occurred before a definitive diagnosis was established, and aggravated after a percutaneous fine-needle aspiration biopsy. Although the bleeding was effectively controlled after TAE treatment, the patient eventually died of aggravated respiratory failure.

Reflecting on this case, we made a series of mistakes during the rapid deterioration of the patient’s condition. Firstly, during the initial consultation, the symptom of abdominal pain was overlooked. Due to a lack of understanding of HSA and its consequent KMS symptoms, the focus of early treatment was on alleviating the patient’s ARDS symptoms and investigating its cause, leading to a waste of precious treatment time. In hindsight, the patient’s abnormal coagulation function and bronchopulmonary hemorrhage symptoms were likely early manifestations of KMS. Secondly, when the patient underwent the first chest CT scan, it reported a space-occupying lesion in the liver, but because the treatment team was still attempting to link the cause of ARDS to a pulmonary infection, this finding was deemed a common hepatic hemangioma and was not further investigated. It was not until the treatment reached an impasse and oncology specialists joined the team. Finally, to obtain a definitive diagnosis, the treatment team had to take the risk of performing a percutaneous biopsy, which, despite thorough preparation, further exacerbated the patient’s condition. The diagnosis of KMS secondary to HSA was then made belatedly by combining the pathological results with coagulation function and clinical symptoms. Prior to this, we had failed to attribute the cause of ARDS to KMS secondary to HSA. However, by that time, due to the patient’s rapidly deteriorating condition, everything had become irreversible. Timely differential diagnosis was missed throughout the entire diagnostic and treatment process. During the initial admission, the patient’s previous liver disease history was erroneously excluded from the etiology due to relatively normal liver function test results. Abdominal discomfort, abdominal pain, poor appetite, and weakness were attributed to infection. During the course of diagnosis and treatment, the coagulation abnormalities were incorrectly interpreted as consumption of coagulation factors due to worsening infection, without considering the potential impact of a neoplastic disease on coagulation function. These 2 instances of erroneous differential diagnosis caused us to lose the opportunity for early aggressive intervention for KMS.

## 4. Conclusions

In this study, we presented a case that exhibited a combination of ARDS and KMS. The diagnosis was misleading and problematic due to rare initial symptoms and presentations. The patient was eventually diagnosed with HSA by pathology and died of unmanageable respiratory failure. Upon reviewing the diagnosis and treatment of this case, it becomes apparent that it represented a diagnostic dilemma. Making a definitive diagnosis based on the patient’s early complex and confusing symptoms caused us great distress, eventually leading to the patient’s death. This case leaves us with several lessons: Firstly, during the initial diagnosis and treatment process, no medical history or clinical manifestation should be overlooked. Even if early symptoms have a very clear indication, we should still carefully analyze symptoms that cannot be explained by the proposed diagnosis, such as the patient’s history of hepatitis and cirrhosis in this case, as well as the persistent abdominal discomfort throughout the treatment process. If the patient’s medical history includes chronic hepatitis, cirrhosis, or previous exposure to hepatotoxic factors, along with digestive system manifestations such as abdominal discomfort, abdominal pain, nausea, vomiting, and poor appetite, early hepatic imaging screening should be conducted to determine the presence of space-occupying lesions in the liver. Dysfunction of a single organ or system may be the external manifestation of a multisystem clinical disease. Therefore, in the clinical diagnosis and treatment process, especially during early diagnosis, while it is important to focus on the primary or typical clinical symptoms, it is equally crucial not to underestimate or ignore accompanying symptoms that lack specificity. When diagnosis and treatment reach an impasse, these “atypical” symptoms often prove to be key in solving the puzzle. Secondly, the patient’s examination results should be clarified as much as possible. For example, the liver mass reflected in the chest CT in this case. If an oncology specialist consultation had been conducted earlier, it might have greatly improved the diagnostic efficiency and prognosis. Lastly, in invasive diagnostic treatments with a high tendency for bleeding, more thorough preoperative preparation is needed to cope with potential rapid deterioration of the patient’s condition. In terms of treatment selection, the patient’s condition should be fully considered, especially coagulation function. Early chemotherapy has the potential to improve KMS, while tumor resection is necessary to fundamentally improve the condition. Although we no longer have the opportunity to apply these treatment modalities to this patient, the combination of preoperative chemotherapy and surgical resection may be the most effective diagnostic and therapeutic approach for such patients.

## Acknowledgments

We would like to express our sincere gratitude to the patient’s family involved in this case report for their active cooperation and selfless contribution during the diagnosis, treatment, and investigation process. Their willingness has allowed us the opportunity to share this case with more researchers to improve clinical diagnostic and therapeutic levels.

## Author contributions

**Conceptualization:** Jiakun Tian.

**Data curation:** Xiaoqian Cui, Min Zhang, Debiao Song.

**Formal analysis:** Xiaoqian Cui, Min Zhang, Debiao Song.

**Investigation:** Xiaoqian Cui, Min Zhang, Debiao Song.

**Methodology:** Xiaoqian Cui.

**Project administration:** Jiakun Tian.

**Resources:** Jiakun Tian.

**Supervision:** Jiakun Tian.

**Writing – original draft:** Xiaoqian Cui.

**Writing – review & editing:** Xiaoqian Cui.
